# Online social transparency in enterprise information systems: a risk assessment method

**DOI:** 10.1007/s10799-021-00347-3

**Published:** 2021-12-22

**Authors:** Tahani Alsaedi, Nada Sherief, Keith Phalp, Raian Ali

**Affiliations:** 1 Department of Computer Science and Informatics, Applied College, Taibah University, Medina, Saudi Arabia; 2 College of Computing and Information Technology, Arab Academy for Science Technology and Maritime Transport, Alexandria, Egypt; 3 Faculty of Science and Technology, Bournemouth University, Poole, UK; 4 College of Science and Engineering, Hamad Bin Khalifa University, Doha, Qatar

**Keywords:** Social transparency, Enterprise social software, Enterprise information systems, Working from home, Covid-19, Teleworking

## Abstract

Teleworking refers to the utilization of information and communication technologies for work done outside the workplace. The Covid-19 crisis led to increased utilisation of social networking tools within enterprises, especially when working remotely. The aim of their use is often to improve situational awareness, coordination, and collaboration amongst employees. Online social transparency, typically done through social networks or enterprise social software, refers to the voluntary sharing of personal and contextual information such as those relating to their own and team status, intentions, motivation, capabilities, goal priorities besides updates on the physical and social context, with other colleagues. An ad-hoc practice of social transparency can introduce risks such as information overload, social loafing and peer pressure. Despite recognising its adverse effects, there is a lack of systematic methods that identify and assess the risks of online social transparency. In this paper, we present a method to identify and evaluate these within enterprises. We present the method’s workflow, stakeholders, the novel artefacts and techniques devised to use and the outcomes to produce. We evaluate our proposed method by applying it in a real organisational context and assess applicability, efficiency, and effectiveness in identifying risks and supporting managers in risk assessment. The results showed that the method gives a framework of thinking and analysis and helps recognize and identify risks in a specialized manner.

## Introduction

At the onset of Covid-19 crisis, several governments called for working from home and recommended companies to facilitate teleworking as an initiative to mitigate the spread of the virus while allowing organisational activities to continue. Companies started to limit in-person interactions and replacing them with telephone and video conferences and started using more kinds of enterprise social software (ESS) to sustain employees engagement [1], enterprise operations, achieve their strategic goals and to mitigate negative economic consequences [2, 3]. To survive in this crisis, an organisation needs to ensure information flow and encourage social transparency between all organizational units and employees; thus, a shared mental model is created upon which team coordination and collaboration is made possible.

Enterprise social software (ESS) is an online platform that allows telecommunication between employees who work remotely to share information about themselves, tasks appointed to them, their progress and collaboration with others [4]. It allows companies to develop their relationships both within the company, between employees and teams [5] and between the organization and their customers [6–8]. These platforms are used to practice social transparency by making the information accessible despite the physical distancing.

Transparency is one of the facets of corporate social responsibilities (CSR) at the organisational level that is intended to drive growth in enterprise performance and productivity [9, 10]. Social transparency is used at the individual level to promote situational awareness, communication, and cooperation between staff and team members [11].

From the perspective of organisational information systems, few works have conceptualized and examined social transparency and its effect on the organisation’s overall performance measure. The authors in [12] advocated that social transparency between the organisation’s employees by disseminating information when someone is engaged in collaborative work would promote the interest of participation and teamwork. They provide a framework for designing conversation-based knowledge communities that support transparency functionalities such as activity and conversation visualization. In [13], the authors state that there are three types of social transparency in online information exchange: (1) identity transparency, (2) content transparency and (3) interaction transparency. They studied the influence of these three types of transparency on the outcome of groups and organisations.

Despite the encouraging implications, the current digital tools and EES seem to be both cumbersome and basic in terms of convenience and efficiency in accommodating useful online social transparency. The ad-hoc manner of utilizing such transparency may generate risks and lead to unfavourable effects such as stress, disturbance, information overload and lack of interest [14]. Research on online social transparency and its side effects is rare in the information systems literature.

Moreover, work on assessing transparency focuses on analysing the design technical issues of user interfaces and navigation facilities [15]. Lourenço et al. [16] developed a model that provides an analysis tool to assess web-based transparency for accountability. The model focuses on three attributes related to online transparency and website technical aspects: visibility, format, and delivery mode. Risks and their assessment, to the best of our knowledge, have not been a primary focus in the literature.

The work in [17–19] showed limitations in the literature regarding assessing the risks of online social transparency:

- Researchers still manage social transparency as a non-functional requirement, and there is a lack of empirical works that address online social transparency as autonomous behaviour by individuals. For example, the authors in [20] considered transparency as a quality requirement for software systems and, therefore, they used modelling tools such as soft-goal interdependency graphs to provide an ontology and reference models for transparency and several quality requirements related to it specifying the interdependencies between them, and determining how to operationalize them.
- Less effort, or probably no effort, is made to systematically identify the negative consequences of online social transparency formally.
- Studies focused on the consequences that stem from information quality, usability, and technical issues, while marginalizing the subject of transparency (the types of disclosed information). For example, the effect of activity and identity transparency on individual and organisational performance is studied in [12, 13].

In the context where employees perform their work remotely or through restricted face-to-face style, a comprehensive framework for the identification and assessment of the risks of online social transparency is necessary to enhance productivity and protect the enterprise from the unwanted consequences of such practice. Thus, in this paper, our aim is to develop a new assessment method to identify and assess the risks and risk factors of online social transparency in enterprise information systems and support enterprise management and system analysts to detect and prioritise risks that stem from unguided conduct of online social transparency. We build our risk assessment method benefiting from the work in [17–19] that applied a multi-stage qualitative study and provided experimental evidence that ad-hoc online social transparency can have unfavourable effects on employees and organisations and, also, provided categories of risks and risk factors. To achieve the aim of our research, we have:

- Identified the gap in the literature of social transparency in enterprise information systems (Sect. 2)
- Explored the assessment factors for online social transparency (Sect. 4.1)
- Explored the risks and risk factors of online social transparency (Sect. 4.2)
- Designed a systematic method to assess and evaluate online social transparency (Sect. 5)
- Evaluated the proposed assessment method for identifying the risks of online social transparency (Sect. 6)

The rest of the paper organised as follows. In Sect. 2, we briefly present background information about online social transparency and the existing approaches for managing transparency. In Sect. 3, we present the research methodology and methods used to achieve the research aim. In Sect. 4, we briefly demonstrate the results of the foundation research work for the assessment method. Then, in Sect. 5, we describe our proposed risk assessment method. In Sect. 6, we describe the case study that we used to evaluate the new proposed method and its results. In Sect. 7, we highlight the key findings. In Sect. 8, we discuss the advantages and application of the proposed method, and present directions for future work.

## Literature review

As social media functionalities in digital devices and internet applications became more integrated into the workplace environment, information about the individual’s identities and their interactions became more visible within and even across enterprise departments. This visibility has been conceptualized as social transparency in the literature of Computer-Supported Collaborative Work (CSCW), Situational Awareness, and Enterprise Management. Social transparency is one of the new research areas that has not been a primary focus in the literature. Social transparency is usually described as conveying social information amongst individuals through online and offline mediums [11, 12]. Therefore, there is always a chance to question the meaning of social transparency in terms of content and purpose and side effects. In this section, we present an overview of previous work on conceptualizing and assessing social transparency and highlight how our work can contribute to bridging the gaps.

### Social transparency in the enterprise: definitions and related effects.

Researchers in [13] defined social transparency as “*the availability of social meta-data surrounding information exchange*”. The authors represented three social dimensions of information exchange that are visible through online applications: *identity transparency*, which refers to information about the people exchanging information; *content transparency*, which refers to the constituents of the content exchanged; and *interaction transparency* which relates to the activities taken during the interaction. In their work on the visualization of work progress in collaborative production context such as open-source software development, the authors in [21] defined social transparency as the visibility of individuals’ activity history which can help others to form an impression of their areas of expertise and to infer and build connections between individuals. They stated that with the increase of transparency about individuals’ actions in the online work context, there is a high potential for leveraging this information to start work relationships and to help recommend people for various tasks.

Social transparency can be practiced through the utilization of enterprise social software platforms (ESSPs) such as Slack, Yammer, and Workplace by Facebook. Kügler, Smolnik [22] highlighted that collaboration, performance, knowledge management, innovation, and employee connectedness to be the areas benefiting most from social transparency of user-created contents (UCCs) in enterprise social software. They defined UCCs as any content shared by an employee, e.g., blogs, text messages, photos, videos, user profiles, and activity streams. Due to its positive impact, transparency is also considered as a factor to measure individual’s collaborative intention to improve knowledge management and knowledge collaboration [23]. Collaborative intentions are described as individuals willingness to share knowledge and how much they want to share their own knowledge [23]. Authors in [12] argued that making co-workers more visible and letting them aware when someone on the team acted on a joint project would encourage participation and promote collaboration work. They discussed three properties of socially transparent systems: *visibility* of social information that enables employees to be both *aware* of what is happening and to be held *accountable* for their actions because of public knowledge of that awareness.

Enterprise members can make inferences about others from what they observe in their work environments. Social transparency in digital work systems has been shown to have an impact on the collaboration amongst enterprise members. For example, the authors in [11] examined the value of social transparency for collaboration in knowledge-based work. They found that social inferences made by individuals based on visible signs of others’ behavior fed into three types of collaborative activities: project management, reputation management, and learning through observation. Authors in [24] argued that making organisational goals and strategies visible and open to employees will make individual performance and contributions to the organisation more evident. Studies in crowdsourcing platforms such as Amazon’s Mechanical Turk found that little or lack of social transparency can lead to psychological distance and reduce motivation to help or perform better [25]. In most crowdsourcing platforms, the identity of task requesters is anonymous, and the purpose and backgrounds of the task are hidden from the workers, which can lead to uncertainty about who they are working for and why they are doing so. This uncertainty has been seen as a factor in producing less passion and commitment to the task.

One of the common impacts of employing social transparency in the workplace is its potential to rebuild trust and reduce reputational risk or damage [26]. The 2010 Edelman Trust Barometer was the first to include transparency and rank it seventh of 16 essential business attributes [27]. Online social transparency has been studied in research that examined the effect of using social media in building social trust amongst peers. For example, Valenzuela et al. [28] stated that using social networks enables individuals to develop norms of trust and reciprocity, which are necessary for engagement in collective activities. However, building trust could be another challenge in teleworking. The level of trust in the virtual team is lower than amongst live-communicating teams [29]. Lack of transparency and trust can become an obstacle to the productivity of the virtual team who face uncertainty and have incomplete knowledge about all team members [30].

Some researchers raised several reasons why they questioned the direct effect of social transparency on accountability. Hale [31] states that accountability is a principle that has two dimensions: the ability to know and the ability to make someone do other things, and transparency and revealing social information can bring the first dimension but not the second dimension. Shkabatur [32] states that the need for accountability is assured by mandatory transparency and reasons that the current transparency policies do not improve liability, also technology has highlighted the drawbacks of transparency procedures. The author illustrated that the current technologies for online transparency enable organisations to withhold some information that might be essential for public accountability. Another reason is the intermediary concept between transparency and accountability. Research on social transparency demonstrated the advantages of the openness about social information between organisational members.

The literature on transparency and its effects highlights the prospective promise of managing social transparency in the enterprise but typically treats social transparency as an issue of information quality. Thus, the clear and structured knowledge about risks and risk factors stemming from the unmanaged behavior of social transparency is still an area that has not yet gained the same attention and empirical investigation. This research aims to fill this gap in the literature.

### Approaches to assess transparency

With the various peculiarities of the concept of social transparency in enterprise and the fine line between its useful and problematic versions, researchers recognized the need for approaches that assess social transparency. It has been already mentioned that the literature lacks a methodical approach to identify and assess social transparency [13] and lacks metrics and measures designed for transparency in the enterprise [33]. Griffith [34] states that systems designers of organisational websites should go beyond the traditional meaning of transparency that can be met by making the information available to those who need them. The need for new criteria for transparency has been emphasized to be able to verify whether it meets users' requirements in the new version of society that appetite for information.

In the empirical studies literature, several investigations have been done to assess the concept of transparency. do Prado Leite, Cappelli [20] examined software transparency through a network of non-functional requirements that need to be comprehensible for both the stakeholders and the software development team. Hence, they proposed a measure for accomplishing useful transparency by identifying its relations with and impacts on other non-functional requirements such as accessibility, informativeness, usability, and auditability. Authors in [35] proposed a framework to capture transparency-related requirements through argumentation. The framework is supported by a language and transparency catalogue. They use the language to create argumentation graphs that represent stakeholders' arguments, conflicts, and preferences about transparency-related NFR. When there is a consensus about these requirements, it is inserted as a requirements pattern in the catalogue. Authors in [11] designed a survey to find an effective way to measure the level of transparency in software development processes. Three attributes were identified in the survey to evaluate the level of transparency: accessibility, relevance, and understandability. The questionnaire aims to identify the problems that may occur amongst stakeholders who involve in the development of software systems. Authors in Hosseini et al. [36] suggested a method that is based on four reference models to administer transparency requirements so that information is communicated in a useful and meaningful manner to its intended spectators. The same authors proposed a modelling language to determine transparency requirements in business information systems [37, 38]. The proposed language includes models to capture transparency requirements in organisations and procedures to argue about the uniformity in the captured requirements.

The literature on online transparency is fragmented and still underdeveloped [39]. Scholars interested in assessing computer-mediated transparency (online transparency) have focused on the content of transparency in websites. For example, a Website Attribute Evaluation System (WAES) has been developed by Cyberspace Policy Research Group and has been used in various research to capture the content of transparency in organisational websites. Some scholars used (WAES) to capture the content of transparency in terms of online availability and technical accessibility[15]. The authors in [40] focused on another dimension of online transparency and proposed criteria that can be used to assess seven objectives of transparency in decision making: accessibility, integration, clarity, logic, accuracy, openness and accountability. Other approaches for assessing internet-based transparency took the perspective of information quality and assessed its timeliness, understandability, completeness, relevance, comparability and reliability [41, 42]. Moreover, an assessment model has been developed to evaluate online transparency with the focus on three attributes which are visibility, format and delivery mode [16]. They stated that these attributes related to information transparency and which are used as essential criteria to assess the information presented in the online websites.

Most of the efforts reported in the literature to assess and evaluate online transparency focused on the quality aspect of transparency. This was to ensure properties such as its freedom from pretence or deceit and being easily detected, apparent and readily understood. We argue that being open and honest may not be enough to be transparent. In this work, we explore a different aspect of transparency which is about explaining reasoning and intentions behind the information shared or actions made. We defined this as social transparency when people are autonomously and voluntarily open about their intentions which underlies any statement they said or action they have done. In conclusion, there is still a lack of methodical approaches to evaluate and assess the quality of online social transparency in general and its risks on performance and wellbeing in specific. In this research, we address the question of how to manage social transparency by first identifying and assessing the risks of its ad-hoc practice.

## Research methodology

The existing literature showed that the available features of online platforms influenced and restricted how enterprises define and utilise online social transparency. Therefore, the first step in our research is to propose a definition of online social transparency to illuminate the concept in a technology-agnostic manner and eliminate confusion with other adjacent concepts. To guide the formulation of our study assumptions and design, we propose the following definition of online social transparency:

*The voluntary use of online platforms by the members of an organisation to share their own information about their situation, roles and responsibilities with other members*.

This voluntary sharing of information can be to improve the quality of situational awareness, coordination, and collaboration among employees in the organisation. Instances of information communicated encompass workload, task priorities, current activities they are involved in, social interdependencies between different team members and employees and their progress and resources, skills’ rank and interest level in specific tasks and/or objectives. In our work, we have the following assumptions:

- Social transparency is practiced voluntarily without any obligation from a higher authority. We do not consider regulatory transparency when people are mandated to be transparent about specific information.
- Social transparency is practiced through online platforms. This assumption excludes the offline and face to face form of social transparency.
- The disclosed information is not essential for others to perform their job and business continuity. This assumption eliminates the functional dependency for informational resources which affects individual ability to play their role and achieve goals and tasks assigned to them [43].
- The disclosed information does not include highly secretive and private information. Privacy is not a prominent concern or a primary factor for individuals in deciding whether to disclose information or not. Instead, factors like disclosing interesting and relevant information in a style that minimizes distraction are more critical

There is a lack of literature on transparency that is practiced voluntarily and its effect on the group work. Therefore, we adopted a qualitative research methodology due to the exploratory nature of this research that aimed first to understand the practice of online social transparency in the daily life of enterprises, the risks associated with it and the assessment factors. Accordingly, we conducted a multistage qualitative study to examine the prospective risks of utilizing social transparency within enterprise information systems. Several data collection methods were employed, as summarized in Table 1. To provide foundations for the risk assessment method, we first worked on: (i) identifying the aspects of the assessment method of online social transparency and (2) investigating the risks and risk factors generated from the ad-hoc manner of practicing online social transparency.

**Table 1:** Research stages, data collection and analysis

Study Stage	Data Collection Method	Purpose
1st Stage: Foundations	Examine related literature including organisational transparency, group dynamics, CSCW, organisational culture, and situational awareness Conducted two focus groups with total 14 participants (scenario-based sessions)	To conceptualize online social transparency To identify key factors for evaluating online social transparency [19]
2nd Stage: Investigation and Elaboration	Conducted Semi-structured interviews with 15 participants	To develop a reference model for the assessment method of online social transparency [17] To identify set of risks and their factors of employing online social transparency within organisations
3rd Stage: Refinement	Observation study Interviews Focus group	To generate a confirmed comprehensive final set of risks and risk factors [18]
4th Stage: Evaluation	Two-phased case study	To validate the proposed method and evaluate its work in real organisational context

All the sessions were audiotaped and transliterated precisely to help in the analysis stage. We followed the six-phase process of thematic analysis suggested by [44]. In the analysis phase, we intended to identify the participants’ viewpoints and their expectations regarding transparency from their colleagues and managers. Also, to identify their problems concerning the effect of this transparency on their positions, social dependencies, and behaviours. The findings of each stage were used as a guide to begin with when analysing the following stage and developed it until we attained the saturation level in all phases. Then we utilized the results of these studies in constructing a new method for identifying and assessing the risks of online social transparency. Finally, we employed a case study methodology to assess our proposed method. All studies were authorized by the research ethics committee of the authors' institution.

All studies were approved by the research ethics board of Bournemouth University. The design of our studies, the supporting material used in them, and samples of what our participants wrote or drafted, can be found in the appendices in [45].

## Findings

### Assessing online social transparency: four basics

**Fig. 1:**
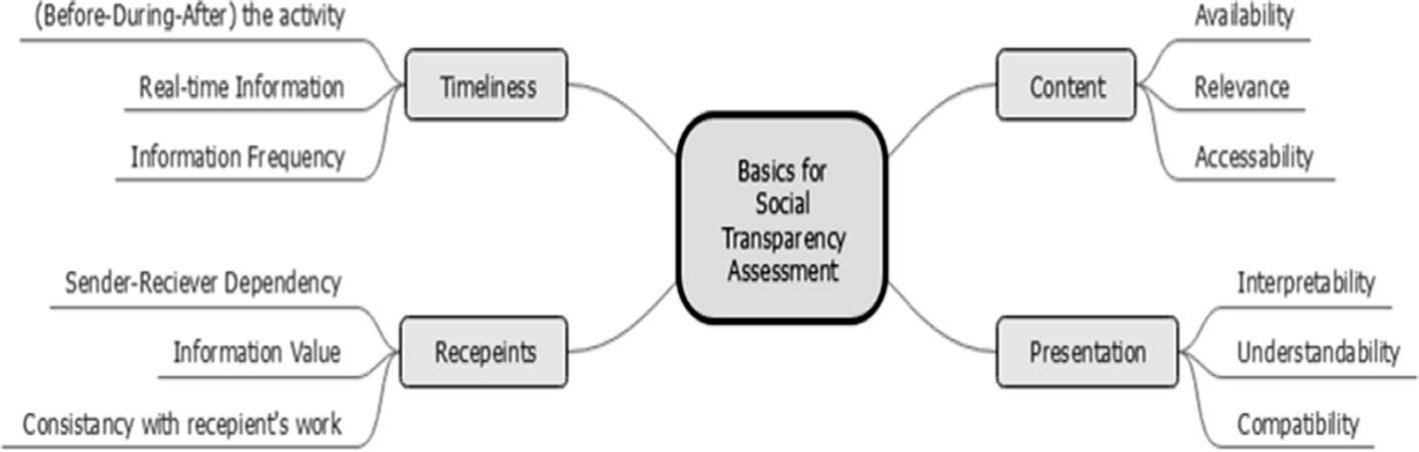
Four basics for assessing online social transparency

The main aim of the first stage of our research is to explore the assessment factors of online social transparency. The findings are presented as a concept map in Fig. 1. This map is intended to help systems analysts as checking points to assess transparency. We found four key factors that play a significant role in evaluating transparency between organisational social actors. The four factors are the content of transparency, the presentation of information, the timeliness of disclosing the information and the characteristics of information recipients [17]. In the list below, we represent these four key aspects. In [17, 19], the risks that are associated with these factors and the importance of including them in the assessment method will be discussed in detail in Sect. 4.2.

• *Transparency Recipients* Transparency should be tailored based on the receivers’ roles and their affiliation with the information providers. The assessment of transparency recipients concentrates on identifying the people who should receive certain types of information and determine the value of the information to them, the dependency among them and other actors, and the consistency of the information with the receiver’s work restrictions.

• *Transparency Content* Transparency allows actors to share information about their activities, which supports others to preserve mental models of their activities and avoid possible clashes. In this context, the assessment methods must comprise inquiries to verify the content of transparency in terms of its availability, importance, and accessibility.

• *Presentation of Information* It describes the degree to which information is well-defined and comprehensible by the recipients [46]. Organisational employees may come from diverse background knowledge, countries, have different education degrees, have different intellectual skills and preferences. The presentation of transparency differs according to the capability of the organisational members to deal with information for their own objectives. We discovered that the content of transparency should be presented in a format that is clear, easy to understand, coherent to the recipients’ capabilities considering their intellectual capabilities and background differences.

• *Timeliness of information* Transparency is only useful when the information sharing is well-timed, which enables the recipients to bring in a positive outcome and get to them when they are prepared and able to make a decision. We found that timeliness of transparency can be categorized relative to the actor’s activity, into five categories: transparency (before-during-after) activity, real-time transparency, and frequent transparency.

### Assessing online social transparency: risks and risk factors

This section presents the findings of the second and third studies that were conducted to explore the various viewpoints of practicing online social transparency in organisational information systems and the potential risks that may occur as a result of its unmanaged practice. Identifying the risk factors is a crucial step in the design of the assessment method to determine and control the level of transparency. As a result of the studies, we discovered four sets of risk factors, and they are interrelated with (1) the provision of information for actors about goals, tasks, and resources, (2) transparency communication (3) transparency sharing practice and (4) the level of transparency. This paper presents examples of the discovered risks and risk factors. Examples of the risks presented in italic and underline text in the following sections. The full details are discussed in [17, 18].

#### Risks and risk factors related to the provision of information

Actors in an enterprise information system represent a dynamic and self-ruling entities that intend to fulfil their goals by cooperating with other actors [47]. They may be human, organisational, or technological entities. In this research, in our studies and analysis we focused on human entities, as individuals or groups. Transparency through online platforms, such as ESS in a collaborative workplace, permits and facilitates to actors the disclosure of information about their demographics, accomplishments, objectives, tasks, utilized resources, and interdependencies. While it usually seeks to strengthen the inter-relationship between actors, we found that this could pose different risks that could lead to adverse effects on their well-being, relationship, and performance. Table 2 shows examples of risks and risk factors identified in relation to the provision of information.

**Table 2:** Examples of risks and risk factors related to the provision of information

Category	Risk Factors	Risks
Actor-related risk factors	Performance	A member in a team may uncover information about issues concerning doing/completing specific tasks to seek out assistance from his/her colleagues, who in turn may take advantage of this information and report to the team leader. This may cause *malevolence* work culture especially amongst actors who have the same level of experience and knowledge to which in turn influences their productivity
	Demographics	Transparency about individual’s information may *cluster* employees in teams with symmetrical members in the level of knowledge or skills. However, the risk of unintentionally disengage employees with the modest level of expertise and knowledge in certain tasks can *diminish their productivity* and may *increase the chance of their turnover*. They may leave the workplace and seek out for places where they can advantage from more professional and highly qualified members
Goal/Task-related risk factors	Status	The absence of transparency about the status of the current task on hand may in the workplace. In the context of collaborative work, lack of knowledge about the task status or progress *increases the level of vulnerability, uncertainty and generate stress* for actors who depend on cooperative goals or tasks, as it becomes more difficult to handle
	Interest	Transparency about an increased level of interest in certain tasks or goal with other members in tasks that require teamwork may trigger risks such as *social loafing* where some members decrease their effort and depend more on those who have high interest to carry out a task
Resource-related risk factors	Outsourcing	Transparency about outsourcing with external parties to provide resources may introduce risks such as *extortion or employee displacement* if the outsourcing is seen as contrary to the enterprise’s culture and standards
	Availability	A lack of transparency about resource availability, especially when needed for exclusive use, may trigger *resource conflict* or overload, which hinders the resource’s ability to serve the execution of the tasks depending on it

#### Risks and risk factors related to transparency communication

The studies in [17, 18] also revealed the significance of evaluating transparency communication as a cross-cutting aspect to the content, recipients, timeliness, and presentation. Table 3 represents examples of risks and risk factors related to this aspect.

**Table 3:** Examples of risks and risk factors related to transparency communication

Category	Risk Factors	Risks
Communication-related risks factors	Relevance Factor	*Information overload* may arise from the transparency of out-of-date information or contradictory information with the recipient’s demands. Moreover, the increased level of *disturbance* and *distraction* in the workplace may result from the practice of irrelevant transparency
	Presentation Factor	Transparency of information that is hard to understand or mismatched with recipients’ requirements may trigger risks such as *loss of interest to collaborate* and *loss of motivation.* Moreover, transparency that involves a high amount of information may *reduce the speed of performance* due to unnecessary time and effort for making a decision
	Timeliness Factor	Due to ill-timed transparency, there could be possible risks of a *delay in progress* and *poor performance*. The lack of timely transparency plays a major role in the workplace's elevated levels of *stress and pressure*

#### Risks and risk factors related to transparency sharing practice

Our studies have shown that risks can be arranged around two main forms of practices of social transparency: asymmetric and symmetric. Asymmetric social transparency occurs when one party is more transparent about his/her information than the other party which makes their perception and knowledge about each other is unequal. Symmetric social transparency identified as the equal transparency behaviour where the two parties are transparent about their information and have enough perception about each other. Examples of risks and risk factors are presented in Table 4.

**Table 4:** Examples of risks and risk factors related to transparency sharing practice

Category	Risk Factors	Risks
Transparency Sharing Practice	Symmetric Transparency	Symmetric social transparency may be the reason of *conditional reciprocity* amongst organisational members. Employees would be socially transparent when their co-workers are transparent too. If their colleagues are consistently unable to be open and transparent, they will have a reputation and other staff will avoid being open to them. This will place *pressure* on staff to avoid losing transparency from others and avoid feelings that arise when they expect colleagues to return to their openness.
	Asymmetric Transparency	The asymmetric practice of social transparency is the imbalance in information sharing between team members. Members who share information, can trigger *power imbalance* as other employees may utilise their information to empower themselves or *abuse the information* for personal gains. Also, blocking information from others is another form of Asymmetric social transparency. It may increase the chance to *weakens group cohesion* due to the asymmetry transparency amongst employees

#### Risks and risk factors related to the level of transparency

The level of transparency shows whether it is sufficient, valuable, or inadequate. The level is fundamentally based on knowledge accessibility, relevance, and interpretability. It is, in other words, a contextual and subjective measure that is determined by the actors and depends on their personal, technical, and social context. We recognized three classifications of the levels of transparency, presented in Table 5.

**Table 5:** Examples of risks and risk factors related to the level of transparency

Category	Risk Factors	Risks
Level of Transparency	Excessive Transparency	Excessive transparency of information associated to a person's work such as status, interest or urgency may result in constructing *information overload* which may lead to *Lack of collaboration*. Due to the excessive amount of information that needs to be addressed, information overload can further *delay the decision-making process*
	Normal Transparency	Transparency of personal skills in resolving certain challenges, either to publicize their abilities or to pass their knowledge to others, may *inhibit the innovation and creativity* of others to discover smart answers and make them more dependent on those who have them
	Lack of Transparency	The lack of clarity regarding the plans of individuals prevents colleagues from understanding the interest of others in some tasks, which can cause a *conflict in the performing of these tasks* and waste significant time on the least priority tasks

## Risk assessment method for online social transparency

This paper intends to present a comprehensive method that supports analysts to evaluate online social transparency in the organisational software and identify its negative consequences. We discussed in Sect. 2.2 the approaches proposed to manage and assess transparency. The focus of the literature was primarily on the management of transparency as a question on managing the quality of information. This paper, however, focuses on the topic of how to scrutinise the negative impact of social transparency by defining and analysing the risks related to when staff apply it in ad-hoc style. The proposed method is specifically designed to employ end-users to identify risks of social transparency in a more structured and organised manner. The risks and risks factors identified in [17, 18] create a part of the method and provide inputs to the analysts and managers to assist them in assessing the risks of social transparency practiced through numerous online platforms in the organisation. Our aim is to enable the enterprises to achieve their strategic objectives more efficiently and at the same time to retain quality and social requirements, such as job satisfaction and a sense of transparency and fairness.

### Theoretical underpinnings of the assessment method

Research on enterprise social computing is primarily driven by the goal of facilitating and governing the process of sharing information and knowledge [48, 49]. However, the means offered for information sharing are not susceptible to the content, presentation, audiences, and interaction time. This implies that risk identification, assessment and mitigation are left for the social actors within the organisation and not handled by systematic approaches and automated tools. Our study identified the need for a systematic method to assess social transparency and prevent potential risks when applying it in enterprise applications. Not like technical enterprise issues that are measured by metrics, voluntary social transparency is a subjective issue, and it is often judgement-based.

There is dynamic nature of social transparency in enterprise apps, which increases its side-effects on organisational members on a daily basis. The objectives of the members of the enterprise may change over time. Decisions on transparency risks and assessments may therefore vary from one actor to another and from time to time for the same actor. [17]. In addition, the regulation of such transparency-related risks can activate a domino effect (i.e., it might introduce another unsought side effect). Based on our studies, we drew the following initial necessities for the intended method to identify and evaluate online social transparency and its risks:

1. *Supporting self-reporting techniques* Smyth, Terry [50] stated that self-report techniques are used to gather personal subjective information that is difficult to be obtained objectively. They also declare that in settings, such as policy making and opinion polls, essential decisions rely on an individual’s subjective evaluation and report of their thoughts and feelings. Therefore, self-reporting techniques such as questionnaires, interviews, or diaries enable enterprise actors to provide information about their thoughts, feelings, behaviours, or experiences of social transparency as a personal and social process.
2. *Supporting a participatory approach* Participatory approach in research has three core principles: empowerment, collaboration, and integration [51]. Regarding the empowerment principle, solutions to social problems rely on the harnessing of the participants and their abilities to experience the problem. From our studies, we found that social transparency is one of the social phenomena in enterprises that is associated with a remarkable self-presentation concern from employee’s sides [17, 18]. Regarding the collaboration principle and reducing the concerns of self-presentation, a method that involves enterprise actors collaboratively in the assessment process should be favourable. This involvement has a high chance of increasing their feelings of ownership, sense of responsibility and their acceptance when using the assessment method and providing their thoughts and feelings regarding the practice of online social transparency. Based on [51], participatory approaches have a dual aim of addressing practical problems and advancing knowledge where action and research are integrated into one single process.
3. *Supporting a longitudinal approach* Social transparency is of a dynamic type, and its side effects change over time, e.g., what could be seen as a useful social transparency for a while, may become redundant and cause information overload when time passes. Therefore, using a longitudinal approach in gathering the data and in the analysis process is proposed. Holland et al. [52] pointed out that longitudinal approaches tend to differ across research disciplines, including, for example, studies in the same community over time, follow-up studies of previous research, recurring interviews with the same participants at regular intervals, and life-course research including data collection across several generations. The following describes the designs factors involved in the assessment method.
  *Data collection over time* The assessment method for social transparency includes the use of techniques such as observation and diary studies over a period of time, as problems may emerge and grow over time and where social contexts play an important role that requires such techniques to capture.
  *Repeated analysis process* The evaluation process must be an ongoing process within organisations to keep the knowledge base current on defined risk factors and threats due to the uncertain and changing nature of the risks of social transparency. A reporting system may be used to convey the discovered risks to managers and assist managers in risk evaluation.
4. *Supporting a detective approach* Enterprises that decide to reduce the risks in their work environment need to identify control activities that can effectively reduce the risk, or the cost associated with them. Control activities fall into three categories: preventive control, detective control, corrective control [53].

Our proposed assessment method in this paper is designed to be a detective method to identify the unremarkable risks and risk factors of online social transparency. Moreover, it encompasses designated risk analysis techniques to enable the decision-makers to investigate and make an informed decision for planning a reduction or prevention solution.

### Steps of the risk assessment method

The argument in [17] was that organisations also need to incorporate social transparency assessment processes that allow for structured governance and policy on the content of transparency, interaction time and audience, without contradicting the innate characteristics of social transparency, mainly its voluntary nature and the need for an organisational culture of openness to allow and empower it. The assessment method should assist systems analysts and enterprise management in planning for risk management strategy. It is expected to help them to identify potential risks that occur because of social transparency through online platforms. To devise this method, several qualitative studies were conducted, including focus groups, interviews, and observational study to capture the underlying concepts and artefacts [17–19]. The assessment method includes two phases: the preparation phase and the action phase, presented in Table 6. The following sections describe the activities of each phase.

**Table 6:** The stages and activities and material to use in our proposed assessment method

Stage	Activity	Description of the steps	Used materials
1. Preparation	1.1 Induction Session	➢ System analysts and management shall conduct induction sessions to inform enterprise staff about •The meaning of social transparency •The potential consequences that may stem from unmanaged behaviour of social transparency •Rationale for analysing social transparency and the need for risk analysis method	➢ Definition of online social transparency (Sect. 1.1 in [45]) ➢ Educational brochure (Fig. 13 in [45]) ➢ Scenarios, which describe the concept of social transparency and context that may cause risks in individual and organisational level (Sect. 7.4.1.1 in [45])
	1.2 Team Creation	➢ Enterprise management calls for volunteers to take part in the assessment process ➢ Creating an assessment team that includes representatives of roles in the enterprise, managers, and systems analysts ➢ Training the assessment team on the observation sheet and goal modelling technique ➢ The assessment team will be provided with a list of risks and risk factors regarding online social transparency	➢ List of risks and risk factors (Table 12 and 13 in [45]) ➢ Observation sheet template (Appendix 1) ➢ Goal model (Fig. 2 in this paper)
	1.3 Training Session	➢ Each member from the assessment team will train a group of staff on using the observation sheet	➢ Observation sheet template ➢ List of risks and risk factors
	1.4 Setting the Analysis Process	➢ System analysts need to build goal model that represents the work boundaries of each role and the strategic dependencies between them ➢ Assessment team, systems analysts and management will collectively identify the following ground rules: •The number of completed observations per individual e.g., at least two completed observation forms by person •The round of assessment process, e.g., weekly, monthly, quarterly, annually	➢ Guidance for goal modelling ➢ Task and schedule sheet
2. Action	2.1 Individual Activity	➢ Staff volunteers shall record and reporting observations by completing the observation sheet	➢ Observation sheet ➢ Definitions of the content of the observation sheet (Sect. 11.5.7 in [45]) ➢ List of risk and risk factors
	2.2 Assessment Team Activities	➢ Reviewing all observation sheets collected from staff ➢ Conducting discussion sessions with system analysts and management to build several analysis charts by using the Goal Model to identify the areas where more attention is needed to minimize its related risks	➢ Goal model ➢ Goal based risk ranking technique (Table 7, 8, and 9 in this paper) ➢ Goal based risk stakeholder Wheel (Fig. 3 in this paper)

#### The preparation phase

In addition to company managers, this stage is expected to be governed by system analysts and will include representatives of each role and position in the organisation. This phase aims to: (1) inform enterprise members about the rationale of the assessment process; (2) decide the participants involved in the assessment process; and (3) set up the scene and the analysis process. This phase involves the following four activities:

*Activity1* Induction sessions to enterprise staff

Enterprise staff need induction sessions to introduce the concept of social transparency, its interactions, platforms and consequences and thus the need and purpose for assessing their social transparency behaviour in the online platforms. The assessment process is based on the voluntary engagement of employees. The importance of induction session comes from the following benefits:

- Several studies in work motivation found that involving employees in the decision-making of their enterprise improvement increases intrinsic motivation and voluntary engagement in the improvement process [54, 55].
- It has been illustrated that engaging employees in the decision-making process make them feel valued from members in ownership and management positions [56].
- By illustrating the power of staff engagement in the success of the assessment process, the chance for efficiently executing the assessment process increases since all staff is committed to the decisions that align with the enterprise values and vision. Self-determination theory is a human motivation theory that linked employees empowerment and their autonomous/intrinsic motivation and commitment in an activity [57, 58].

System analysts and enterprise management can support the induction session by collecting some information from research findings and organise them in educational materials such as a brochure or generate scenarios from the work environment to provide examples of situations where risks might occur and identify their factors**.** Educational material helps to deliver the information and immerse the staff in the context of the problem. Examples of educational materials can be found in [45].

*Activity 2* Creation of the assessment team

This activity aims to create a multi-faceted team that can assess the risk of social transparency in the enterprise. To be able to assess the risks, the team must first be able to identify the vulnerabilities in the enterprise systems and various types of risks and risk factors that can exploit those vulnerabilities. Creating the assessment team and preparing them includes two steps.

*The first step is recruiting the team* members by setting the responsibilities of the assessment team and then advertising for volunteers from representative roles in the enterprise, system analysts, and management members. The voluntary recruitment also involves employees that are not in the level of system analysts and managers. The reasons for involving enterprise employees in the assessment process are: (i) they know more about the enterprise culture which consequently maximize their abilities in identifying the vulnerabilities its related risks, (ii) saving the time that may be spend in training an external team on the enterprise culture, policies, and structure, and (iii) ease of accessibility since the management can reach the assessment members when it is needed and the assessment team can also reach the enterprise staff, teams and departments.

*The second step is training the assessment team* to prepare them for the assessment process and develop their ability in using the analysis materials. The training course will include a description of the following materials that will be used in the assessment process:

- *A list of risks and risk factors* In [17, 18], we explored various factors that are considered as main sources of risks that stem from the unmanaged practice of social transparency. These risks and risk factors are presented inTables 2, 3 and 4 in Section 4.
- *Observation Sheet* A novel artifact**,** an observation sheet, has been designed to collect the data from the enterprise staff. We proposed an observation sheet as a human-centred approach that allows such diversity to be collected in a more organised way, given the type of data and the individual differences in risk assessment. Our findings discussed in [17, 18] were used to design the observation sheet which can be found in Appendix 1 to match the particularities and specific nature of social transparency risks and the risk factors.
- *Goal modelling* is one of the well-known approaches that represent the why behind the enterprise architecture in terms of rationale, goal, and requirements [59]. Social transparency is a behaviour that occurs amongst staff to express their thoughts, feelings, and commitments towards their work, including their goals, tasks, and resources. As a result, the assessment process of social transparency will utilize the goal modelling approach as a baseline to provide a clear visual presentation of the enterprise social system and its activities. In [45], section 7.4.1.4 elaborates more the reasons of integrating the enterprise goal model into the assessment method. In the assessment method, the goal model is used in two different risk analysis techniques (i.e., goal-based risk ranking technique and goal-based risk stakeholder wheel).

*Activity 3* Training all enterprise staff members

Each member of the assessment team will play the role of a trainer to train a group of staff on how to use the observation sheet and the rationale for using observation sheets to assess social transparency. The trainer will also use the observation sheet template that can be found in the appendix, the vocabulary definitions of the sheet (Sect. 11.5.7 of [45]) and list of risk and risk factors to illustrate how observation should be provided. It is important in this step to exemplify and clarify for the staff what kind of information should be considered as social transparency.

*Activity 4* Setting the analysis process

This activity aimed to prepare the assessment team for the analysis process in the next phase (i.e., Action phase). The analysis of the risk factors can be guided by the utilization of the goal modelling approach as a baseline to provide a clear visual presentation of the enterprise social system and its activities [60]. In this step, the assessment team alongside system analysts will build the goal model of the assessment environment, including the actors, their goals, tasks, soft-goals and interdependencies between them. This step is required to effectively assess the impact of identified risks, which will be explained in the following section.

Also, in this activity, the assessment team is required to set the common ground rules to articulate a set of expected behavior for staff participation in the assessment process. The assessment team alongside the enterprise management should collectively decide the following ground rules:

- The number of observations that should provide per person e.g., at least two observations per person
- The period for providing the observation, e.g., per week, bi-weekly, or monthly.
- The round of the assessment process, e.g., weekly, monthly, quarterly, or annually.

Setting ground rules play an important role in providing positive results from the analysis process and prevent issues from occurring that can interfere with the assessment process such as lack of participation that leads to lack of sufficient inputs needed for accurate risk identification and assessment. The assessment team must remind staff about the ground rules periodically, particularly if problems occur in the enterprise, for example, delays in achieving short-term goals.

#### The action phase

The second phase of our proposed assessment method is the risk identification and risk analysis process. This phase aimed to (1) provide actionable information from observation data; (2) determine the risks and the factors that cause their occurrence; (3) highlight the areas of social transparency that need more attention. This phase involves the following activities:

*Activity 1* Collecting the observations from staff members

In this step, staff members are requested to provide observations and encouraged to use the ground rules set in the previous step for providing observations. This step is based on voluntary participation from staff to report their concerns regarding the social transparency of their peers. We argued that risks of social transparency are unanticipated in the workplace. Therefore, a technique such as voluntary self-reporting enables staff to provide information about their thoughts, feelings, or experiences of social transparency. Assessing social transparency is a new quality assurance procedure in the workplace, which makes staff hesitant to engage in the assessment process and provide observation. Thus, the anonymity of providing the observations is important in overcoming the resistance of the staff to express their true concerns about undesired behavior of social transparency.

*Activity 2* Analysing the collected observations

This step is the core actioning step in the assessment method. In this step, the assessment team identifies and analyses the risks that appear in the enterprise social system. The observation sheet proposed in this method has been designed as an input means to inform reasoning about employees’ goals, tasks, and interdependencies. In this work, we developed two novel analysis techniques to be generated collectively based on the use of enterprise goal model. These techniques have been deployed from the risk management process which they are (1) goal-based risk ranking technique and (2) goal-based risk stakeholders’ wheel.

*I. Goal-based Risk Ranking Technique* is a technique that can be used by the assessment teams to evaluate the severity of the identified risks in the work environment. This can be performed by evaluating the impact of their occurrence on certain tasks and goals captured through a goal model. In risk management, there is no one single way to determine the level of the risk. Risks ranking requires the knowledge of the workplace activities, the urgency of the situations, and objective decision. For simple situations, an assessment can be a discussion or brainstorming session based on participants knowledge and experience. In other cases, checklists or a probability matrix can be helpful. For more complex situations, a team of knowledgeable professionals who are familiar with the work and tool support are usually necessary. In our method we propose that with the use of enterprise goal model, the assessment team and enterprise management would be able to rank risks and organise them in various impact levels. In Table 7 we classify the impact on goals and tasks into four levels.

**Table 7:** Risk impact levels

Risk Level	Description
Catastrophic	The risk has a major effect on enterprise productivity in terms of quantity and quality and requires urgent actions. For example, lack of collaboration and engagement due to lack of transparency
High	The risk has a significant effect on the enterprise productivity in terms of quantity. For example, social loafing in collaborative tasks
Critical	The risk has a minor effect on the enterprise productivity and needs action to improve the system. For example, information overload due to excessive transparency
Marginal	The risk can be avoided by individual strategy. For example, stress that stems from a certain task can be avoided by trying one of the alternatives of that task

In the proposed assessment method, activity refers to either a goal or a task that is influenced by the occurrence of certain risks. We assume that activity is represented by one role, without consideration of individual instantiation and differences. Table 8 explains the guidelines to identify the risk impact based on the occurrence of the specified activity properties.

**Table 8:** Risk impact based on goal modelling activity properties

Risk Level	Activity Properties
Catastrophic	If activity has a strong contribution to a soft-goal If activity has no alternatives If activity has dependency from another task/ goal/ resource/ soft goal If activity is part of an AND decomposition, i.e., mandatory
High	If activity has no alternatives If activity has dependency from another task/ goal/ resource/ soft-goal If activity is part of AND decomposition, i.e., mandatory
Critical	If activity is part of OR decomposition with one alternative If activity has dependency from another task/ goal/ resource/ soft-goal
Marginal	If activity is part of OR decomposition with more than one alternative If activity has no dependency from another task/ goal/ resource/ soft-goal

The goal-based risk raking technique can be summarized in the following steps to be followed by the assessment team.
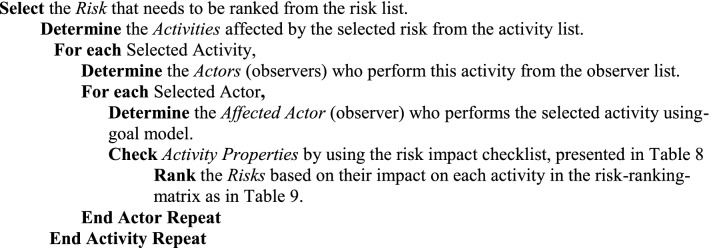


The assessment team uses the template presented in Table 9, to visualize and organise the impact of the risk of each activity. Some risks may have different impacts that occur in two different activities. This analysis technique helps enterprise management to make an informed decision to plan for a mitigation process according to risk prioritization. Decisions of the impact of the risk rely on a discussion amongst the assessment team and enterprise management because they know well the enterprise strategy and, which activities have a high impact on enterprise productivity.

**Table 9:** Risk ranking matrix

	Activity 1	Activity 2	Activity 3	Activity N
Risk 1		Catastrophic		High
Risk 2		Marginal	Catastrophic	
Risk 3			Marginal	
Risk N	Marginal	Catastrophic		

*II. Goal-based Risk Stakeholders’ Wheel* Stakeholders’ wheel is one of the techniques that is used to determine the direct and indirect stakeholders affected by a change. We note that actors are inter-dependent within enterprises, and this means risks can propagate as it does in traditional supply chains. Therefore, we developed the stakeholders’ wheel to represent the direct and indirect stakeholders that are influenced by the occurrence of certain risks. The design of the stakeholder wheels is simple, the assessment team put the risk in the middle of a circle and surround it with directly affected stakeholders (1st level) and then surround the first level with the indirectly affected stakeholders (2nd level, 3rd level, …). Figure 3 in Sect. 6.3.2 shows an example of one of the stakeholders’ wheel that built in the evaluation study.

The assessment team, with assistance from system analysts, can use the ranking risk template and goal model to create a goal-based stakeholders’ wheel as detailed in the following steps:
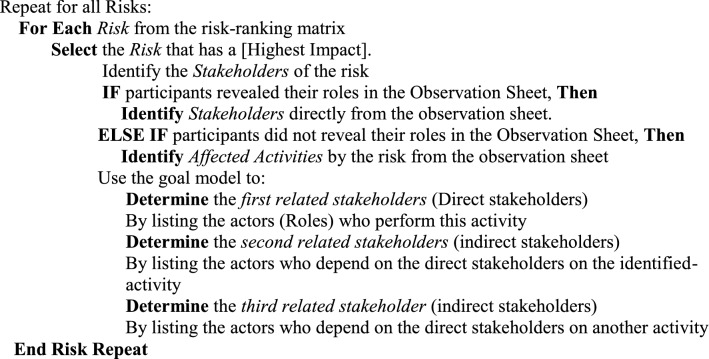


Once the stakeholders’ wheels for all risks are completed, the assessment team can get a visual overview of the direct and indirect stakeholders who may be influenced by the occurrence of the identified risks, their origin, impact whether on activities or stakeholders.

## Evaluation of the assessment method

This section describes the two-phase evaluation study that we conducted to assess the extent to which the proposed method provides an enhanced customization method that aids system analysts and management in assessing online social transparency and detecting the potential risks and their factors. It also aims to examine the usability of the assessment method and its supporting materials in terms of understandability, comprehension, effectiveness and helpfulness.

### Evaluation method

For the evaluation stage, a case study approach was adopted to evaluate the proposed assessment method in a real context. The case study approach is defined as “a strategy for doing research which involves an empirical investigation of a particular contemporary phenomenon within its real-life context using multiple sources of evidence” [61].

For the nature of the social nature of transparency and its risk assessment, the case study is the appropriate approach to evaluate the proposed assessment method of online social transparency. Moreover, a case study evaluation helps to determine how the assessment process could help practitioners in determining the risks and their impact in their real work environment. The aim of the evaluation was threefold:

- Examining the effectiveness of the assessment method in detecting the risks of online social transparency from a real work environment
- Examining the ability of the assessment method to support managers in assessing the identified risks and to facilitate the collaborative decision-making process for risk mitigation planning,
- Examining the applicability of the assessment process to be adopted in a real work environment.

The evaluation study has been conducted in a non-profit educational organisation based in Alexandria, Egypt. This organisation supports different activities in the fields of engineering, business science, and technology. The mission of this organisation is to offer comprehensive educational programs, training, and consultancies. It is considered as a large organisation with more than 6000 employees across 8 branches in 3 countries. Employees use emails, Facebook, and WhatsApp for social interaction with their colleagues, teams, and managers. Eight participants play the role of the assessment team for their organisation. Participants’ details are summarized in Table 10. The assessment team involved volunteered employees who play a facilitator role in gathering information from organisational members and analyst role in assessing the risk and risk factors from the collected information with system analysts and managers. Inclusion criteria required that participants have knowledge about risks identification and risks assessment. All system analysts and managers have experience in software engineering and systems analysis. The managers have a proficient level of experience in risk identification and risk analysis.

**Table 10:** Participants details in the evaluation study

Participant no	Role in the organisation	Role in the evaluation study	Size of Supervised Team	Years of experience
1	Head of Quality Assurance Unit	System analyst and manager	–	15
2	Software Architect in the Information Centre	System analyst and manager		12
3	Head of the college website maintenance committee	System analyst	–	11
4	Head of the college scheduling committee	System analyst	–	11
5	Office director	Facilitator	5	10
6	Teaching assistant	Facilitator	4	8
7	Teaching assistant	Facilitator	4	8
8	Teaching assistant	Facilitator	4	6

### The evaluation study procedure

In this section, we explain the procedure of the evaluation study of the proposed assessment method, which is summarized in Table 11. The evaluation study involved the following sessions:

**Table 11:** Procedure for the evaluation of the assessment method

Induction Session:	*Aim* The session aim was to introduce:
	• Social transparency
	• The risks and risk factors
	• The research problem
	• The study aims
	• Ethical considerations
	• Lasted about 30 min
	• Employees and system analysts involved
Assessment Sessions:	*Aim* The sessions aim was to identify and assess the risks of social transparency in the organisation:
	• Phase 1: With the aid of the proposed method
	• Phase 2: Without the aid of the proposed method
	*Phase 1* the session aim was to identify the risks and risk factors of social transparency without the aid of our proposed method using:
	• The list of risks and risk factors
	• The traditional risk acquisition methods
	• Manual analysis of risk impact and risk stakeholders
	• Lasted about two hours
	• Employees and system analysts were involved
	*Phase 2* aimed to identify the risks and risk factors of social transparency with the aid of our proposed method. This phase involved two sessions:
	*Training Session*:
	• Trained the facilitators on using the observation sheet
	• List of risks and risk factors
	• Lasted about 30 min
	• Trained 4 Facilitators
	• Facilitators were given 10 days to collect observation sheets from employees
	*Analysis Session:*
	• Introduced the concept of goal modelling and its notation
	• Built a goal model for the participants' workplace
	• Analyzed the collected observation sheets
	• Ranked identified risks using goal-based risk ranking technique
	• Analyzed the effect of risks on stakeholders using goal-based risks stakeholders’ wheel
	• Lasted about four hours
	• Facilitators, systems analysts, and managers were involved

#### Induction session

The two types of participants (i.e., employees and system analysts) engaged in the same induction session to introduce the research problem and the aim of the study. Then, we introduced the concept of online social transparency and its negative consequences by providing relevant examples and scenarios that show the risk areas of unmanaged practice of online social transparency amongst employees. The induction session was held for about 30 minutes.

#### Assessment sessions

These sessions aimed to identify and assess the risks of online social transparency. These sessions are divided into two phases:

*Phase 1* This phase aimed to identify risks of online social transparency *without the aid of our proposed assessment method*. Therefore, in this phase, both types of participants, i.e., the volunteer employees and systems analysts were involved in this session to detect and analyze the risks of online social transparency based on the techniques used in their organisation. This session lasted 2 hours. At the beginning of the session, the researcher introduced types of risks and risk factors that may stem from the unmanaged practice of online social transparency. The purpose of this step is to bring the subject to discussion and to make it notice in their online interaction practice. Examples of such risks and risk factors were discussed earlier in this paper, and details can be found in [17, 18].

After introducing the previous concepts, the researcher aimed to identify the current techniques they use in risk identification and assessment to identify their pitfalls and gaps through the following questions: As a member of the assessment team of online social transparency in your organisation,

1. How would you identify the risks and risk factors of online social transparency in your workplace?
2. How would you evaluate the impact of the risks on the work environment?
3. How would you rank the risks of social transparency (i.e. based on which metrics)?

*Phase 2* This phase aimed to identify the risks and risk factors of online social transparency and evaluate their impact on the work environment *with the aid of our proposed assessment method*. This phase involved the following two sessions:

- *In the first 10-day study*, facilitators were trained to use the proposed observation sheet, and were provided with a list of risks and risk factors. As detailed in section 5, our assessment method is designed to engage employees to gather information about the potential risks of online social transparency. Thus, the facilitators were asked to distribute the observation sheets in their workplace and asked employees to fill them voluntarily. Facilitators were given 10 days to provide the collected observations from the employees. Total of 17 observation sheets were collected. During the demonstration of the observation sheet, the researcher took notes of the facilitators’ enquiries and questions as an evaluation of the observation sheet that will be discussed in Section 6.3.2.
- *In the analysis session***,** facilitators, system analysts, and managers were asked to use the data collected from the first 10-day study to identify and assess the impact of the risks with the aid of the proposed assessment method and goal-based risk analysis techniques. As preparation for this session, (1) we introduced the concept of goal models and its notation and (2) we built a goal model for the participants’ workplace. In this study, the goal model presents one college in the educational organisation. The researcher engaged in this session as an observer for clarity and understandability purposes. This session lasted for 4 hours. At the end of the session, participants were provided with a survey to evaluate the assessment method and its supporting materials (i.e., goal-based risk ranking and goal-based risk stakeholders’ wheel), which will be discussed in Section 6.3.

### Case study results

Throughout each phase, all participants were promoted to think aloud to verbalize their thoughts while working through each of the evaluation activities. The sessions were audio-recorded and transcribed verbatim for analysis purposes. The following sections describe the results for each phase.

#### Results of phase 1

This phase has been conducted to investigate how the assessment team, including employees, system analysts and enterprise management can (1) identify the risks of online social transparency and (2) assess and prioritize the risks based on their effect on the work environment by *using the current techniques used for risk assessment in their organisation*. At the beginning of risk identification activity, the participants were asked to write a list of social software used in their organisations to enable the participant to recall examples from this software and to link the risks to their sources of online platforms. The participants suggested traditional techniques that may help in identifying the risks of social transparency and their factors, such as interviews, lesson learned from previous experience and questionnaires. The participants were asked to provide a list of the risk and the risk factors that may result from the practice of social transparency using their social software. Some participants suggested conducting an interview with all employees or distributing a questionnaire because risk may differ from one person to another. However, in this session, they provided their answers based on a discussion of their previous experiences. Examples of the findings are presented in Table 12.

**Table 12:** Examples of risks identified by using traditional methods

Risk	Risk Factor	Description
Information overload	Irrelevant information	Transparency of information in the general chat rooms that involve all employees. There was a consensus that information overload is the most common risk of social transparency. It was pointed out that *“lack of instructions about transparency practice in the group chat room ends up with sharing irrelevant information”,* which leads to information overload
Distracting from work	Frequent and Instant transparency	Distraction may happen due to involving in several chat rooms. Some participants who has more than one role in the organisation stated that might be a member in several chat rooms such as chat group with employees from same department, chat room with quality assurance team or chat room for examination team. Random and frequent transparency about each employee’s updates can cause distraction for other members
Minimum commitment	Task interest	In collaborative task between course lecturer and teaching assistant such as marking, transparency of less interest to perform the task may reduce the commitment from collaborators

The risk identification and analysis in this organisation is part of the quality assurance department. They require reports from all employees about their courses and they analyze these reports manually to identify the sources of weaknesses and faults. During the risk analysis and assessment activity, participants were asked if they think that information about stakeholders’ activities and dependencies is useful for risk assessment, identifying the impact of risks and how would they utilize this information.

From their perception of the concept of social transparency and its potential risks that introduced at the beginning of this session, there was a consensus on the importance of using this information in identifying the impact of the risks on the organisational members, their activities, and their relationships. Some system analysts declared that they currently use techniques for conducting biannual risk assessment. For example, assessment for higher-level courses by mapping all college’s courses to the formulated student outcomes to ensure the student outcome attainment and to identify the stakeholders who may affected by any noted risks. They stated that they currently use organisational charts that show the hierarchy and the dependency between roles to detect the roles that may affected by the identified risks. They also use narrative description to document the responsibilities of each role. They argue, *“They can detect the impact of the risks on the specific activity and also detect the dependencies and propagation of risks through the analysis of both documents”*. However, they argued that the currently used techniques for risk assessment require time and effort to reach a decision.

It was noted in this activity that participants were struggling to find a systematic way to analyze the impact of the risks based on the organisational structure particularly the impact on the stakeholders’ activities and dependencies. Some system analysts suggested extracting the risks by interviewing employees and identifying the direct actors who has reported these risks. Then linking the identified risks with those actors by using the organisational chart and tracing the roles that has dependency with the direct actors and might be affected by these risks. However, they claimed that a better encapsulated representation will help in the analysis as well as a systematic way is needed to accurately link the risk with the actors’ activities and identify the actors who may affected by this risk.

The discussion in this phase highlighted several barriers that prevent participants from adequately identifying and prioritizing the risks of online social transparency in their work environment. These barriers are discussed in the following six points as follows:

- *Lack of conceptual clarity* It was stated during the discussion that risk identification techniques used in their enterprise are designed to detect risk in specific problems such as problem-related to the quality of the teaching courses, course progress, course exams and student withdrawal from certain courses. These risk identification and risk assessment are designed using well-described conceptual frameworks for educational systems. However, the participants illustrated that risk identification for behavioural problems is not a well-known process across the organisation and using the same identification methods is not applicable. Therefore, the understanding of central concepts such as risks, risk factors, vulnerability, and checking points substantially varies between employees, departments, companies.
- *Difficulties in collecting the data* One of the issues that were declared in the session is the difficulty in collecting data related to the risks of social transparency. This difficulty is explained in the following two points: (1) The first point relates to the *difficulty of collecting data in large-scale organisations*. Thus, to waive such a problem there is a need for more committed and experienced roles with well-defined tasks to do the data collection process; (2) The second point relates to the *unstructured manner of gathering the data*. From an employee's point of view, there are no guidelines to help them provide the needed information.
- *Difficulties in interpreting the data* One of the obstacles noted by observing the participants in this session was the description and interpretation of the collected data. The collected unstructured data needs to be revised by analysts and presented in a formal manner to obtain useful and meaningful information that facilitates the analysis process. Also, the participants claimed that there is a lack of procedure for transforming the raw data into structured, useful and meaningful information used to enable more effective decision-making. They argue that a lack of structured representation of the results may be *“discouraging the decision-makers from the assessment of social transparency”.*
- *Difficulties in identifying reliable and accurate risks* Risk identification in this phase relied on the participants’ prediction and their individual experiences in the consequences of social transparency. Some participants stated that their current assessment process might not be effective due to the unreliability of the identified risk factors extracted from unstructured data interpreted in a subjective manner. They agree to the need for identification techniques that extract reliable and accurate risks from real situations.
- *Difficulties in linking risks with models of organisational structure* It was noticeable that the participant has difficulties in analysing the impact of the risks based on models of organisational structure such as organisational charts and roles description. There were some attempts to link the risks to the activities and the dependencies between the actors, but these attempts can be complex and require time and effort due to the unstructured format of the collected data and also the textual formats that contain the roles and responsibilities of the staff members. Therefore, the participants need a procedure that enables them to analyse the impact of the risk and link it to the organisational model.
- *Lack of technical capacity* It was stated that the process of collecting data from employees (i.e. by using interviews) in large companies might generate a wide range of big data records. The system analysts participate in this study have no knowledge about assessing social behaviour in the work environment. Therefore, they stated that their company lack qualified analysts in such kind of problems, which will need training. Some participants suggested contracting experts in organisational behaviour for this purpose. However, it was argued that there are a few possibilities to apply this suggestion in some companies, due to the cost of time and money to contract with experts.

#### Results of phase 2

This phase aimed to evaluate the use of the proposed assessment method and its artefacts to identify and assess the risk of online social transparency of the social software used in the enterprise. During these sessions, the researcher played the role of observer. The session was audio-recorded and transcribed verbatim for further analysis. As preparation for this phase, facilitators were trained on how to use and fill the observation sheet. Each facilitator was responsible for training a group of employees who are willing to participate in the study. The employees were given 10 days to provide their observations. A total of 17 observations were collected from the employees. There was a general positive agreement that the observation sheet was one of the useful artefacts used in the proposed method. It provided several advantages as specified by the participants:

- *Ease of use* there was a general positive agreement that the sheet was well structured. The detailed structure and supporting key terms (labels) of the sheet was the advantage that helps the employee to complete the sheet. It was stated that the instruction section in the sheet was helpful for users to fill the sheet effectively. A participant described this section as “a reference for the users”. Involving this section in the observation sheet enables the users to remember the steps and conditions when it is needed.
- *Language used* The participants were from different roles, skills, and levels of experience. They stated that the sheet was written in simple language that can be understandable to all employees. However, some participants had to return to the definitions document (i.e. educational materials provided in the preparation phase) of the sheet to ensure correct understanding of some terms. Also, the differentiation between the observer the person who experienced the risk, and the observee, the person who caused the risk was confusing to them and a suggestion was made to clarify that in the instruction section.
- *The length of the sheet* Collecting the data in the proposed assessment method was based on the voluntary participatory approach from organisational members. Therefore, one of the criteria that was important to us designing an observation sheet that is acceptable and does not require a long time to be filled. Some participants stated that *“The first time was the longest time to complete the sheet”* due to the unfamiliarity with the terms and questions at the first time. As a result of the learning effect, it was commented that the employee spent less time on subsequent ones.
- *Helpfulness of supporting materials* The observation sheet was attached with a document that can be used as a reference if the participants face difficulties in understanding the meaning of some terms in the observation sheet. The document was structured as a glossary that contains definitions of all terms in the sheet and provides descriptions and examples of these terms. It was stated that *“the structure of the definition document is divided based on the sections of the observation sheet which enable the users to find the intended section easily”*.

**Fig. 2:**
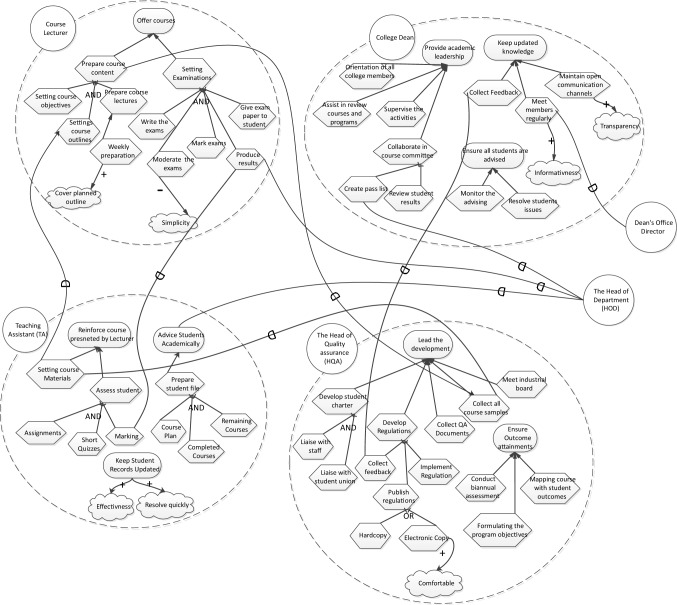
Part of the organisation goal model built in the study

Next in this phase, participants were asked to analyze the data collected from the employees by observation sheet to (1) identify the risks and risk factors, and (2) assess the impact of the identified risks based on the organisational goal model. As preparation for this activity, a goal model was built for their workplace, as presented in Fig. 2. Then, the participants were given a list of questions and tasks to guide the discussion during the session.

The session started by reviewing and reading the observation sheets to familiarize themselves with the collected information. The participants suggested starting of thinking for a way to present the vital information in the sheet in a well-structured report.

Based on their experience on risk analysis, the participants suggested organising the information in a tabular format to present the identified risks, their factors, the used platforms, the person affected by the identified risk and how many required actions to solve the problem. There were various risks identified, such as missing activity, pressure, delay in progress, conflict of goals, loss of motivation and task quitting compared to what was identified using traditional methods. Table 13 presents a sample of the identified risks. A full version of the identified risks can be found in Sect. 8.4.3.2 in [45].

**Table 13:** Sample of risks identified by analysing the observation sheets

Risk	Information type	Risk factor (s)	Online platform	Affected employee	Action needed	Suggested actions	Quote	Concern Level
Goals conflict	Goal-based information	1. Lack of transparency about the goal 2. Information not provided after goal achieved 3. Observer depends on observee to achieve goal 4. Both collaborate in the same goal 5. Both located in the same workplace 6. No equal transparency between them	WhatsApp	Information center staff	No	Clarity of ranking and rewards from the management	“The information of a goal was hidden by the observe to achieve and rank higher than his colleague”	High
Missing activity	Social	1. Lack of transparency 2. Information provided after activity3. Both employees located in the same workplace	WhatsApp	Office Director	No	Provide clear answers	“Due to bad weather, the organisation president decided that only 10% of the staff present. The college dean of was not transparent about who he needed to be present at the college. So, I thought I am not going to be included in the 10% who can present. Accordingly, I missed the college board meeting”	Medium

After identifying the risk and risk factors, the participants were asked to assess the impact of the identified risks by using the organisational goal model they built in Fig. 2. There are two goal-based risk analysis techniques designed to assess the impact of the risk: (1) goal-based risk ranking and (2) goal-based risk stakeholders’ wheel. The participants were provided with a description of the analysis techniques and their steps to facilitate the assessment process.

Following the steps of the goal-based risk ranking technique detailed in Sect. 5.2.2, participants chose to start with stress that was reported by the teaching assistant (TA) in the activity named “Project discussion”. By using the risk severity criteria described in and goal model in Fig. 2, the severity of stress has been categorized as high because project discussion activity has no alternatives, and it is part of AND decomposition and the course lecturer depends on the TA to perform this activity and provide the marks as analyzed from the goal model in Fig. 2. The same steps have been done on the other identified risks. Table 14 shows the generated risk ranking matrix.

**Table 14:** Generated risk ranking matrix

	Project discussion (Task)	College board meeting (Task)	Present Lectures (Task)	Maintain an updated version of course outline (Task)
Stress	High			
Delay in progress				High
Information misuse			Catastrophic	
Missing activity		High

**Table 15:** Summary of the advantages and suggested improvements of the assessment method

Dimension	Pros	Suggestions
Understandability	Well-structured format Reasonable amount of details	Add recommendations and best practices template to identify the maximum number of members in the assessment team and how often the assessment will be repeated (i.e., weekly, monthly, and annually)
Comprehensiveness	Materials were a reference for the participants to clarify the contexts and the situations that suitable for the purpose of the assessment process	Some amendments have suggested to be made in the document of the risk matrix. The risk ranking technique might need to separate the risk in relation to tasks from the risk associated with goals for obtaining better insights into the effect of the identified risks on the organisational structure It was also suggested to augment the risk ranking and risk stakeholders’ techniques with some steps to organise the selection of the risks to implement these techniques
Effectiveness	Observation sheet was a creative method to extract the risks from real situations Goal-based risk analysis techniques were the essential additions that improve the effectiveness of the assessment method	Implementing the method and analysis techniques in automated format to facilitate the assessment process and save time and effort in the decision-making process and to produce risk ranking and risk stakeholders automatically
Helpfulness	The helpfulness of the assessment method to identify and prioritize the risks The discussion during the implementation of these techniques shows the usefulness of the participatory approach in supporting the decision about assessing the risk impact The engagement of employees from various roles in the assessment method accelerates the discussion regarding the value of specific activity and dependency from other roles	Participants required adding more statistics about the correlation between risks and their factors as well as a visual presentation for the risk factors

**Fig. 3:**
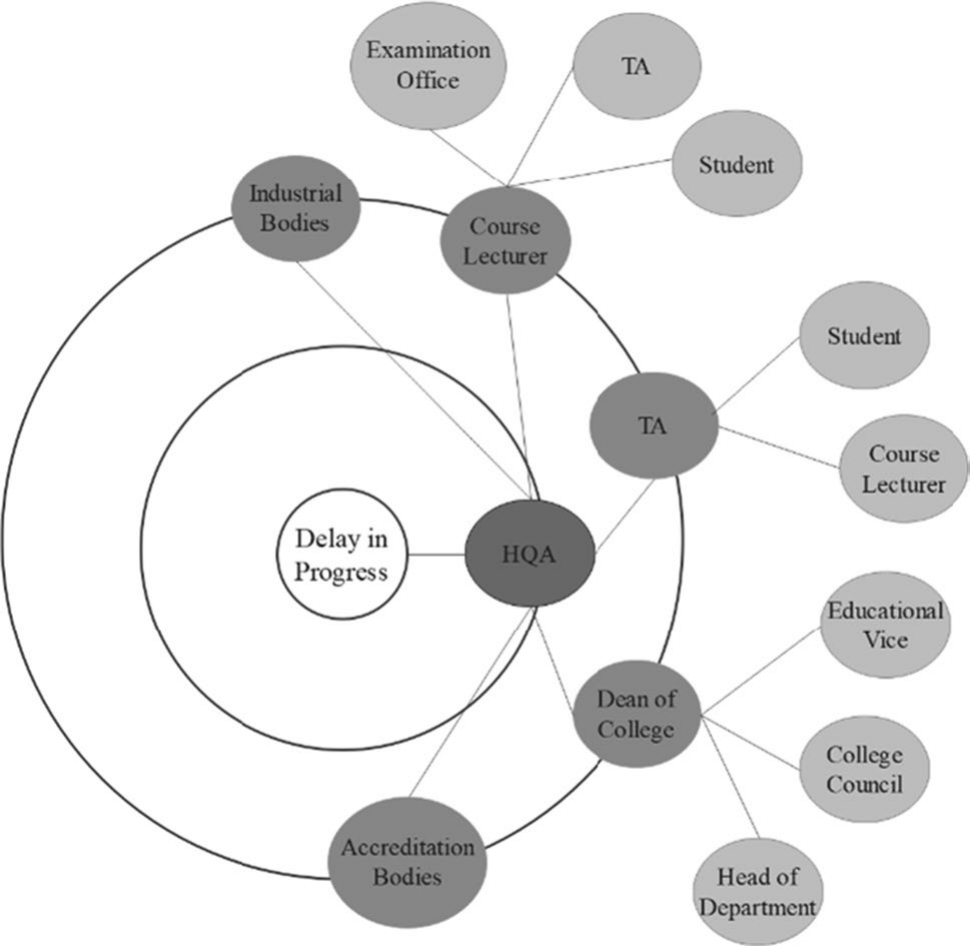
Generated goal-based stakeholders’ wheel

In the goal-based risk stakeholders’ wheel, the participants followed the steps of the method to identify the direct and indirect stakeholders that may influence by the occurrence of this risk as described in Sect. 5.2.2. For example, they started with Delay in progress risk that occurs in the activity “Maintain an updated version of course outline”. This activity is part of the head of the quality assurance goal’s model (HQA). This role classified as the first stakeholder influenced by this risk. Then they track the roles that have a direct dependency with HOD and the roles that also has a dependency on those direct dependers. Figure 3 shows an example of the goal-based stakeholder wheel generated in this activity.

The discussion in this session raised some points that are considered necessary for the efficiency and helpfulness of the assessment method. The following are the points observed in this activity.

- It was stated that the way of linking these analysis techniques with the goal model is novel in the risk analysis process. Some system analysts have a good knowledge of these techniques due to the nature of their work in risk assessment. They advocated the importance of linking risk analysis techniques with the organisational model to clearly examine the potential impact of the risks on the organisational activities.
- In the risk ranking technique, It was noted that participants spent a long time deciding which risk they should start with. The description of the techniques is missing clear criteria of the risk that should be assessed first. These criteria were left to the assessment team to decide the risk priority based on their impact on the work environment.
- A system analyst stated that the assessment method could be overwhelming if it implemented manually, especially with a large number of observations.
- In the risk ranking technique, the columns represent the activities reported by employees in the observation sheet. However, the activity may refer to task or goal. Participants suggested two ways in solving the clarity of this matrix: (1) adding the type of activity in brackets after writing the name as seen in or (2) creating two separate matrixes, one for tasks and one for goals. It was argued that organisation usually concern about achieving a strategic goal, then creating two matrixes provide a clear insight into the individual goals that may adhere to the achievement of the organisational goal.
- Some points were essential to increase the efficiency of the goal-based risk analysis techniques. It was suggested to add weight in the goal-based risk stakeholders’ wheel to identify the impact on the work environment. The stakeholders present a role that may be played by more than one person. It was emphasized that adding the number of people who play specific roles may help understand the impact of the identified risks.

*Quality criteria for the assessment method*

The aim of the evaluation study is to assess the extent to which the proposed method aids system analysts and management in assessing online social transparency and detecting the potential risks and their factors. It also aims to examine the usability of the assessment method and its supporting materials in terms of the following four aspects: Understandability, Comprehensiveness, Effectiveness and Helpfulness. Table 15 summarizes the pointed advantages of the assessment method and suggestions to improve it.

*1. Understandability* It refers to the aspect that the assessment process and its supporting materials are presented in a way that makes it easy for users to understand them. This aspect was necessary for the evaluation of the assessment method. Understandability can involve several aspects such as clarity, concision, and structure. During designing the assessment method, we intended to provide a clear and straightforward method with less complicated details that can be readable and understandable from people with different knowledge about risk analysis.

- The evaluation study involved participants from diverse backgrounds and experience in system analysis and requirement engineering. It was noted from the evaluation sessions and the survey’s answers that there is a consensus that the assessment steps in was described in a well-structured format with a reasonable amount of details. One participant described the content of the assessment method as “seamless and straight to the point”. This structure helps the participants to follow the steps for each activity and predict the needed outcomes for the next activities. A manager recommended adding a recommendations and best practices template in the outcome of activity 4 (Setting the analysis process) in the preparation phase to identify the maximum number of members in the assessment team and how often the assessment will be repeated (i.e., weekly, monthly, and annually).
- Regarding the supporting materials, including goal-based risk analysis techniques, participants’ answers show that the description of the supporting materials clarified how to use them. A participant stated that “the technique was user-friendly” and “do not require high knowledge in analysis.” System analyst commented, “The content in the technique description was reasonable to understand the whole results in one setting.”

*2. Comprehensiveness* It refers to the aspect that examines the assessment method in terms of comprehensive the completeness of the explanation of its activities, the supporting materials, the description of the roles involved in the assessment process and their responsibilities, and the prerequisite knowledge needed for using the method.

- The materials were in the form of documents that include a list of risk and risk factors, a description of the terms in the observation sheet and guidelines for the goal-based risk analysis techniques. In regard to these documents, it was generally stated that these materials were a reference for the participants to clarify the contexts and the situations that suitable for the purpose of the assessment process.
- There were suggestions to improve the completeness and quality of the assessment method. As mentioned before that the participant suggested adding a policy template in the outcome of activity 4 in the assessment framework. Some amendments have suggested to be made in the document of the risk matrix. The risk ranking technique might need to separate the risk in relation to tasks from the risk associated with goals for obtaining better insights into the effect of the identified risks on the organisational structure. It was also suggested to augment the risk ranking and risk stakeholders’ techniques with some steps to organise the selection of the risks to implement these techniques.

*3. Effectiveness* This aspect represents the ability of the assessment method and the supporting materials to identify, prioritize, and assess the risks of online social transparency. The effectiveness of the assessment method and the supporting materials has been compared with the traditional methods used for risk analysis such as interviewing. The evaluation has shown a general satisfaction with the effectiveness of the proposed method in identifying and assessing the risks not only in terms of the number of generated cases but also in terms of yielding more accurate results that can be used for better analysis.

- It was advocated that the use of an observation sheet was a creative method to extract the risks from real situations. The observation sheet has been an improved way to support the accuracy of the identified risks. It has been argued that interviewing might be another way of detecting risks from real context, but it may rely on the recall of the previous situations while the observation sheet can detect risks from a real-time context.
- The goal-based risk analysis techniques were the essential additions that improve the effectiveness of the assessment method. After performing the identification and assessment of the risks with and without the supporting materials, there was a general endorsement that these materials were useful in answering participants’ enquiries. However, a participant suggested implementing the method and analysis techniques in automated format to facilitate the assessment process and save time and effort in the decision-making process and to produce risk ranking and risk stakeholders automatically. This suggestion will be considered in the future work of this research.
- A system analyst declared that the success of these techniques relies on the quality of the information provided in the observation sheet. It was stated *that “the technique would work well if the employees explicitly provided a full description of their roles, the activities and stick with one risk per observation sheet”*. The integration of some conditions in the observation sheet will help the employees to provide valuable information that facilitates the assessment process.

*4. Helpfulness* It is an aspect that presents the ability of the assessment framework to provide help and benefits to the assessment team. The main aim of designing this framework is to help the organisations to assess the real implementation of online social transparency in their work environments. The risk of online social transparency is unremarkable in these organisations. It was stated that the assessment framework enables *“the management to track personnel who are under threats due to online social transparency”*. Therefore, the evaluation study approves that the assessment method and the supporting materials were helpful in recognizing the risks that are found in the work environment and their factors that cause the occurrence of these risks.

- The helpfulness of the assessment method was not just about the ability to identify and prioritize the risks; system analysts pointed out that visual presentation of the results is required to understand trends and gain general insights about the collected observations. Participants required adding more statistics about the correlation between risks and their factors as well as a visual presentation for the risk factors.
- The goal-based techniques were designed to be implemented manually and based on the discussion amongst the assessment team. The discussion during the implementation of these techniques shows the usefulness of the participatory approach in supporting the decision about assessing the risk impact. The engagement of employees from various roles in the assessment method accelerates the discussion regarding the value of specific activity and dependency from other roles. Participant from management stressed the importance of engaging various roles in the planning stage for the mitigation process. It was stated that “having employees in the assessment process accelerates the determining of risk stakeholder.”

## Discussion

In remote work situations like those faced during the Covid-19 pandemic, organizations needed to sustain employees engagement and maintain their ability to communicate, collaborate and function properly [62]. Therefore, there is a need for various engagement practices to help support employees working from home. Examples of these practices are kids engagement while their parent work from home [63], invest in communication tools and recognition platform that help employees to send and receive recognition [64], keep transparent communication between employees, solicit their feedback and freely share information both frustration and idea with colleagues for development in a productive way [65]. Despite the advantages of practicing transparency amongst employees, the unmanaged practice of transparency may introduce unexpected and unfavourable consequences as discussed in [17–19]. The findings of this research revealed the need for a structured and systematic assessment method to identify and assess the risks associated with the ad-hoc practice of transparency in workplace and help organization management to avoid unpleasant results.

Most the approaches for managing the practice of transparency in enterprise information systems are designed without the consideration of the negative consequences (i.e., risks) of an ad-hoc practice. Findings in our previous works [17, 18] demonstrated the need for designing a systematic method to monitor social transparency in the enterprise and assess its negative impact on stakeholders and work environment. Online social transparency has been researched in several works from the technical aspect of social software, i.e., the features of the social software. These works investigated the regulatory aspect of the concept. However, this research focuses on the subjective aspect of online social transparency and its correlation with organisational model, particularly sharing intentions and personal reasoning. Unlike technical organizational issues that are assessed by metrics, transparency on a voluntary basis is a subjective issue, and it is often judgement based.

While some approaches were proposed in the literature to manage transparency in information systems as discussed in Sect. 2.2, risks and risk factors were not their focus. Therefore, this research is meant to help in enriching the area of online social transparency by proposing new dimensions that could help system analysts and designers in providing elements for developing the implementation of online social transparency within the organisational settings. The conducted studies in this research resulted in exploring risks, risk factors and a set of assessment factors needed in designing a comprehensive method to evaluate the impact of online social transparency [17–19].

Our analysis shows that social transparency has an unpredictable nature, and its side effects evolve on the daily life of employees. It also shows that employees’ goals may change over time. Therefore, we found that decisions regarding transparency risks and assessment can vary from one actor to another and in the same actor from time to time. Thus, we argue that traditional risk identification methods such as interviews, scenario-based method and expert checking may cost time and effort as well as may not reflect an accurate vision of the whole situation. This paper introduced a systematic method for identifying and assessing risks of online social transparency. This assessment method involved a novel risk identification method (i.e., observation sheet) designated to collect risks and risk factors based on individual observation. The proposed risk identification method was designed to reduce the assessment cost and time for enterprises that have a high number of employees and resources. In addition, this identification method enables the access to a wider and diverse set of enterprise members and contexts that might be unpredictable by using traditional techniques. For example, in using scenario-based techniques to predict the risk of social transparency, it cannot be certain how employees’ impressions about the situation introduced in the scenarios. Similarly, Interviews can be used to collect data from real enterprise members, but it might take a long time to cover all employees in the enterprise. The proposed assessment method was designed to keep up with the rapid nature of social transparency and allow all the enterprise members to provide up-to-date information about undesired social transparency daily and with short amount of time. This helps in maintaining the analysis results up to date.

To examine the effectiveness of the proposed assessment method, we evaluated it using a case study of a real organization to examine the usability of the assessment method and its supporting materials in terms of the understandability, comprehensive, effectiveness and helpfulness. The evaluation study utilised a think aloud protocol in two phases. Phase 1 providesd insights on how the traditional identification and analysis techniques may not be appropriate to assess the risks aspect of online social transparency and the need for a well-designed method to identify the risks and prioritise their impact. Phase 2 provides insights on how the users would use the proposed assessment method and its dedicated material to effectively identify and assess the risks of online social transparency. In this phase, we identify the amendments that may help in improving the usability of the proposed assessment method. The same participants engaged in the two phases. The evaluation study gave us a multi-faceted understanding of what system analysts and management expect from the decision support method to assess social behavior. However, there were certain situations that were counteracted by the researcher to avoid the threats that may affect the validity of the study. For example, the selection of the participants was based on personal connections with the researcher. This kind of sampling may affect the trustworthiness of the answers. People are yet aware of the risk of social transparency, and they concern that discussion of this topic may affect the general impression of their organisations. Therefore, we select participants who already have a trust relationship with them. In addition to the sample selection, the time given to the participants was limited. This could affect the quality of their performance and discussion as raised by some of them. However, the engagement of experts and experienced analysts were helpful in accelerating the discussion, particularly decision in the goal-based risk analysis techniques. However, the open-ended survey was designed to overcome this limitation and enable the participants to freely provide their insights and suggestions in the proposed assessment method and its supporting materials.

## Conclusions and future work

In this paper, we proposed a comprehensive, staged method to assess online social transparency that helps decision-makers to be aware of its risks and risk level in their organisations. There are several contributions in this paper. First, we presented empirical evidence that online social transparency is linked to risks and that it requires structured and systematic techniques for diagnosis and assessment. Second, this paper added to the corpus of knowledge by offering a detailed framework for identifying and assessing the major elements that might lead to workplace social transparency risks and negative consequences. Third, an observation sheet was invented to obtain replies from employees. It gives a method for systematically identifying risks in a real-world setting on a regular basis. This unique identification technique is the foundation stage of the proposed system. It not only offers a systematic way of recording daily observations, but it also adds value by assisting employees in delivering accurate observations and system analysts in accurately identifying risk and risk factors in real-world scenarios.

Although the proposed assessment method has shown its ability to detect and assess the risks of online social transparency, participants were still concerned about the time and effort required to perform the assessment process in regular basis which may affect on the acceptance to perform the proposed assessment method. This issue can be solved by proposing an automated version of the assessment method by using business intelligence features. As future work, we suggest providing a tool that collect data, analyze, monitor, and visually display the important assessment results and allow the assessment team to interact with the data and enable them to take well-informed and data-driven decisions. The assessment tool can benefit from the integration of several techniques that used in recommender systems such as information reusability, risk prioritization and investigation for missing information. We also suggest conducting further studies to propose several mitigation strategies that can help decision makers to link the risk impact with an appropriate strategy.

## Rights and permissions

This article is licensed under a Creative Commons Attribution 4.0 International License, which permits use, sharing, adaptation, distribution and reproduction in any medium or format, as long as you give appropriate credit to the original author(s) and the source, provide a link to the Creative Commons licence, and indicate if changes were made. The images or other third-party material in this article are included in the article’s Creative Commons licence, unless indicated otherwise in a credit line to the material. If material is not included in the article's Creative Commons licence and your intended use is not permitted by statutory regulation or exceeds the permitted use, you will need to obtain permission directly from the copyright holder. To view a copy of this licence, visit http://creativecommons.org/licenses/by/4.0/.

## Data Availability

The design of our studies, the material used in them and samples of what our participants wrote or sketched, can be found in the appendices of [45].

## Abbreviations

ESS: Enterprise Social Software
CSR: Corporate Social Responsibilities
CSCW: Computer-Supported Collaborative Work
ESSPs: Enterprise social Software Platforms
UCCs: User-Created Contents
WAES: Website Attribute Evaluation System
FoMO: Fear of Missing Out
TA: Teaching Assistant
HQA: Head of the Quality Assurance
HOD: Head of Department
